# Local Twitches During Ultrasound-Guided Fascial Hydrorelease Occur Within Stacking Fascia: A Retrospective Analysis of a Large Video Archive

**DOI:** 10.3390/medsci14030350

**Published:** 2026-06-27

**Authors:** Hiroaki Kimura, Tadanao Hiroki, Tadashi Kobayashi, Hideaki Obata

**Affiliations:** 1 Kimura Pain Clinic, Maebashi 371-0013, Japan; 2 Department of Anesthesiology, Isesaki Municipal Hospital, 12-1 Tsunatori-honmachi, Isesaki 372-0817, Japan; t-hiroki@gunma-u.ac.jp; 3 Development of Community Healthcare, Hirosaki University Graduate School of Medicine, Hirosaki 036-8562, Japan; tkoba@hirosaki-u.ac.jp; 4 Department of Anesthesiology, Saitama Medical Center, Saitama Medical University, Kawagoe 350-8550, Japan; hoobata@gmail.com

**Keywords:** fascia, local twitch, stacking fascia, fascial hydrorelease, ultrasound-guided procedure, retrospective analysis, anatomical selectivity, hyaluronic acid, densification

## Abstract

**Background/Objectives**: Ultrasound-guided fascial hydrorelease (FHR) occasionally elicits a brief localized contraction (“local twitch”) at the moment the needle tip contacts a fascial layer; the anatomical basis of this reaction has not yet been systematically characterized. To examine local twitch occurrence relative to stacking fascia (yes/no) at the needle tip (primary outcome), as well as the anatomical distribution and per-video capture rate (secondary outcomes). **Methods**: We retrospectively analyzed 11,205 ultrasound videos from a single pain clinic (October 2015–March 2026). Twitches were identified by prospective clinical observation and computational screening (frame-difference-based Profile Match classifier; 417 candidates over 30 review rounds). The stacking fascia status was independently determined by two FHR-experienced clinicians, with discordant cases jointly adjudicated. **Results**: Inter-rater agreement was 86/90 (95.6%; 95% CI 89.0–98.8%); one case was reassessed, deemed to not be a twitch, and excluded. In the final cohort (n = 89), local twitches occurred at stacking fascia in 89/89 (100%; 95% CI 95.9–100%). Events were concentrated in gluteal/pelvic (51%) and lumbar paraspinal (29%) regions, with a per-video capture rate of 0.98% (110/11,205; 95% CI 0.81–1.18%). **Conclusions**: Local twitches during ultrasound-guided FHR essentially always coincide with the needle tip lying within stacking fascia, identifying this as the structural locus within this cohort. This figure represents inclusion-criterion-bound selectivity within the twitch-positive subset, not the positive predictive value of stacking fascia for twitch occurrence.

## 1. Introduction

Ultrasound-guided fascial hydrorelease (FHR) has been developed over the past decade as a therapeutic intervention for chronic musculoskeletal pain [1]. During routine FHR, clinicians occasionally observe a brief, localized contraction (“local twitch”) on the ultrasound image at the precise instant the needle tip contacts a fascial layer. The twitch appears abruptly as a single discrete contraction lasting less than 1 s; it is spatially restricted to the tissue immediately surrounding the needle tip and does not recur unless the needle is withdrawn and re-inserted. Although this phenomenon has been recognized clinically for years, its incidence, anatomical distribution, and structural substrate have not been systematically characterized. The absence of systematic characterization reflects two practical difficulties: the phenomenon is rare on a per-procedure basis, and definitive identification requires simultaneous ultrasound visualization of the needle tip and surrounding fascial structure. These constraints have hitherto limited investigation to anecdotal reports.

Clinical experience indicates that local twitches during FHR preferentially occur at “stacking fascia,” an ultrasound-visible phenotype of densified, multilayered deep fascia characterized by fascial adhesion and gliding impairment that appears as a hyperechoic band-like lesion [1,2]. Stecco and colleagues have shown that fascial densification results from hyaluronic acid aggregation and increased ground substance viscosity [3,4,5]; this molecular–architectural account is consistent with broader characterizations of the hyaluronan content of fascial connective tissue [6] and with the active contractile properties recently demonstrated in deep fascia [7], an interpretation supported by detailed histological studies of the deep fasciae [4]; stacking fascia can be interpreted as the ultrasound-visible phenotype of this densified state. In previous work, we proposed the “Fascial Memory Reset Hypothesis”, in which increased tissue stiffness activates YAP/TAZ mechano-transduction signaling and, through mechano-epigenetic regulation, establishes a “fascial memory” that maintains the pathological fascial state; FHR may reset this memory over days to weeks [2]. The structural–molecular basis of stacking fascia has been increasingly characterized in recent fascia research. The deep fascia comprises layered collagen sheets separated by hyaluronic-acid-rich loose connective tissue, with specialized cells called fasciacytes regulating the gliding properties between these layers [8]. Recent work has further elucidated the molecular composition of human deep fasciae [5] and the role of mechanotransductive signaling pathways such as YAP/TAZ in fascial cells [9]. The Fascial Memory Reset hypothesis, recently proposed in this journal [2], positions stacking fascia as a structural phenotype of densified deep fascia maintained by mechano-epigenetic regulation, and frames fascia hydrorelease as a procedure capable of resetting this pathological state on a mid- to long-term time scale. Against this background, the immediate-phase mechanisms by which fascia hydrorelease elicits its clinically observed effects—including the local twitch response (LTR) explored here—remain incompletely understood. Candidate mechanisms span mechanoreception by muscle spindles and other proprioceptive endings [10,11], classical motor-endplate-mediated twitch responses described in the trigger-point literature [12,13], and biophysical phenomena specific to multilayered collagen-hyaluronan architecture. The central observational question addressed here is whether local twitches are structurally specific to stacking fascia—that is, whether densified multilayered fascia represents the anatomical substrate at which twitches are generated.

The primary aim of this study was to characterize the frequency of local twitches and their fascial-layer selectivity through large-scale retrospective analysis of ultrasound videos recorded during routine FHR practice. This study serves as the observational anchor of our research program on ultrasound-guided FHR. Accordingly, this observational study is deliberately limited to anatomical and imaging-level questions—where and under which structural conditions local twitches occur—while mechanistic interpretations are only briefly outlined and are developed in detail elsewhere.

## 2. Results

**Figure 1:**
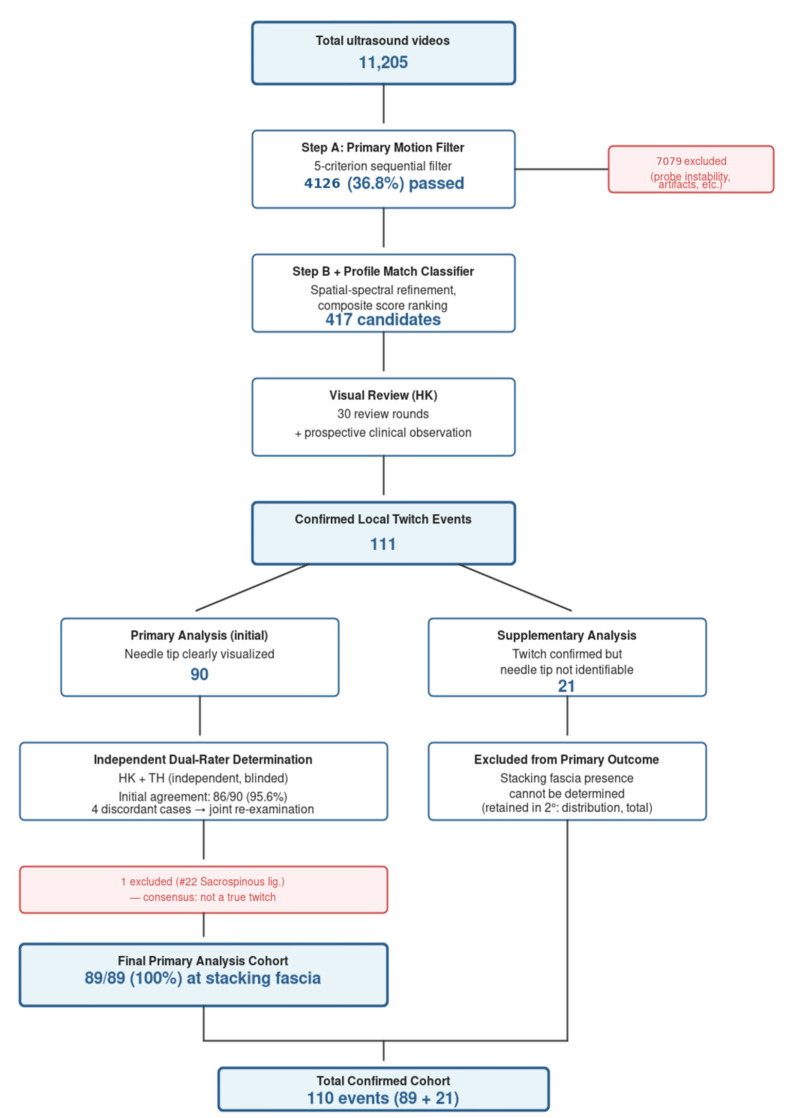
Computational screening and determination flowchart. The two-step computational pipeline (Step A primary motion filter; Step B + Profile Match classifier) reduced 11,205 ultrasound videos to 417 candidates that underwent visual review across 30 rounds. Combined with prospective clinical observation, 111 events were confirmed: 90 (initial primary analysis) + 21 (supplementary). Independent dual-rater determination yielded an initial agreement of 86/90 (95.6%); 4 discordant cases were resolved by joint re-examination, with 1 case (#22 sacrospinous ligament) excluded as not a true twitch event. Final primary cohort: 89/89 (100%) at stacking fascia. Total confirmed cohort: 110 events.

**Figure 2:**
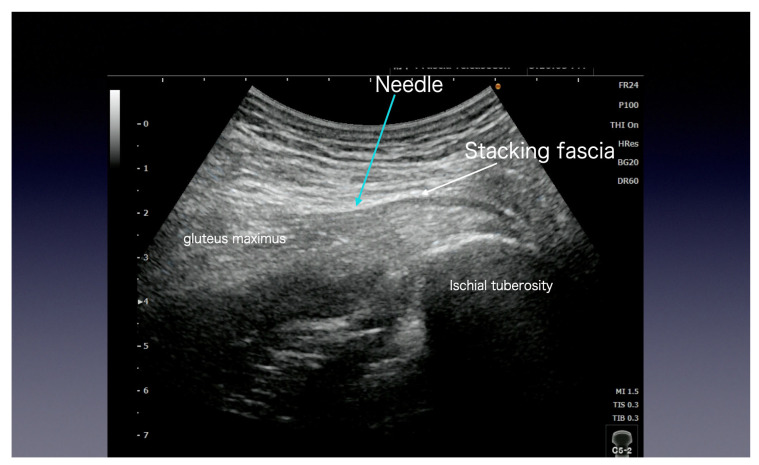
Representative ultrasound image of a local twitch event during fascial hydrorelease (FHR). The needle tip (cyan arrow) contacts the stacking fascia (white arrow) between the gluteus maximus and semimembranosus, with the local twitch centered on the stacking fascia. Acquired with a Konica Minolta linear probe (C5-2/L18-4, FR 24, P 100, THI On, HRes, BG 20, MI 1.5, TIS 0.3, TIB 0.3).

This Results section is organized as follows. We first describe the video dataset and overall screening outcome (Section 2.1; summarized in Figure 1 and Table 1), then report the primary outcome of stacking-fascia presence at the needle-tip site (Section 2.2), the secondary outcomes of incidence and anatomical distribution (Section 2.3), and the quantitative motion characteristics of the confirmed events (Section 2.4; Table 2). The overall study design and the computational screening pipeline that identified candidate local twitch events from the video archive are summarized in Figure 1, and a representative ultrasound image of a local twitch is shown in Figure 2. Representative local twitch videos are shown in Supplementary Videos S1–S3.

Figure 2 shows a representative ultrasound image of a local twitch event captured during fascial hydrorelease, with the needle tip contacting stacking fascia at the moment of contraction. This example illustrates the characteristic anatomical configuration that motivated the systematic, large-scale analysis reported in the following subsections.

### 2.1. Video Dataset and Cohort Definition

A total of 11,205 ultrasound videos recorded between October 2015 and March 2026 (approximately 10 years and 6 months) were analyzed. Overall dataset characteristics—including recording period, consent framework, ultrasound equipment, clinical indication, and computational screening statistics—are summarized in Table 1. Consistent with the retrospective, de-identified nature of this video-based analysis, individual patient-level demographic data (age, sex, body mass index, and symptom duration) were not systematically extracted from clinical records and are therefore not reported here; the cohort reflects the spectrum of chronic musculoskeletal pain patients treated at a single pain clinic over approximately ten and a half years, predominantly presenting with low-back, gluteal/pelvic, and cervicoscapular pain. Of the 11,205 videos in the archive, 4126 (36.8%) passed automated screening based on image quality and probe stability (Step A; see Table 1); the remaining 7079 videos (63.2%) were excluded for one or more of the following reasons: excessive probe motion or operator hand-shake, suboptimal image depth or gain settings, recording artifacts, and frames in which the needle was not visualized for a sufficient duration to support frame-difference analysis.

### 2.2. Primary Outcome: Stacking Fascia (Yes/No) at the Needle-Tip Site

Stacking fascia determination was performed independently by two FHR-experienced reviewers (H.K. and T.H.). The first author (H.K.; >10 years of ultrasound-guided FHR experience) determined whether the needle tip was at stacking fascia for each of the 111 confirmed twitch events. The second reviewer (T.H.) subsequently performed an independent determination of the same 111 events, without access to H.K.’s determinations. Initial inter-rater agreement on the primary-analysis cases was 86 out of the 90 (95.6%; 95% CI 89.0–98.8%) at stacking fascia. The four discordant cases were resolved by joint re-examination of the ultrasound video clips with shared reasoning between the two reviewers. Of these four, one case (sacrospinous ligament site, slow muscle contraction lasting several seconds) was reassessed by both reviewers as not constituting a genuine local twitch event (likely a voluntary muscle movement) and was excluded from the primary-analysis cohort. The remaining three (obturator internus, piriformis, and lumbar facet joint) were confirmed by both reviewers as twitches occurring while the needle tip lay within stacking fascia. The final primary analysis cohort thus comprised 89 cases, in all of which (89/89, 100%) consensus placed the needle tip within stacking fascia at the moment of the twitch. In the 21 supplementary cases, the twitch event itself was clearly observed on ultrasound, but the needle-tip position could not be reliably identified; the presence of stacking fascia at the needle-tip site could therefore not be determined. These cases were retained in the secondary outcomes (anatomical distribution and total event count) but were not used for primary outcome assessment.

### 2.3. Secondary Outcomes: Incidence of Local Twitches and Anatomical Distribution

As outlined in the introductory overview (Figure 1), the screening cascade and case-selection funnel underlying every subsequent analysis is the following. From the 11,205 ultrasound videos, 111 local twitch events were initially identified through the combined clinical observation and computational screening phases (Profile Match classifier, in which 417 candidates were visually reviewed across 30 review rounds). Of these, 21 cases with unclear needle-tip visualization were assigned to the supplementary analysis, and 90 cases with a clearly visualized needle tip constituted the initial primary analysis set. Following independent dual-rater determination (Section 2.2), one case in the primary-analysis cohort (sacrospinous ligament site) was reassessed by consensus as not constituting a true local twitch event and was excluded; the final primary analysis cohort therefore comprised 89 cases. The overall final per-video capture rate was 0.98% (110/11,205).

**Figure 3:**
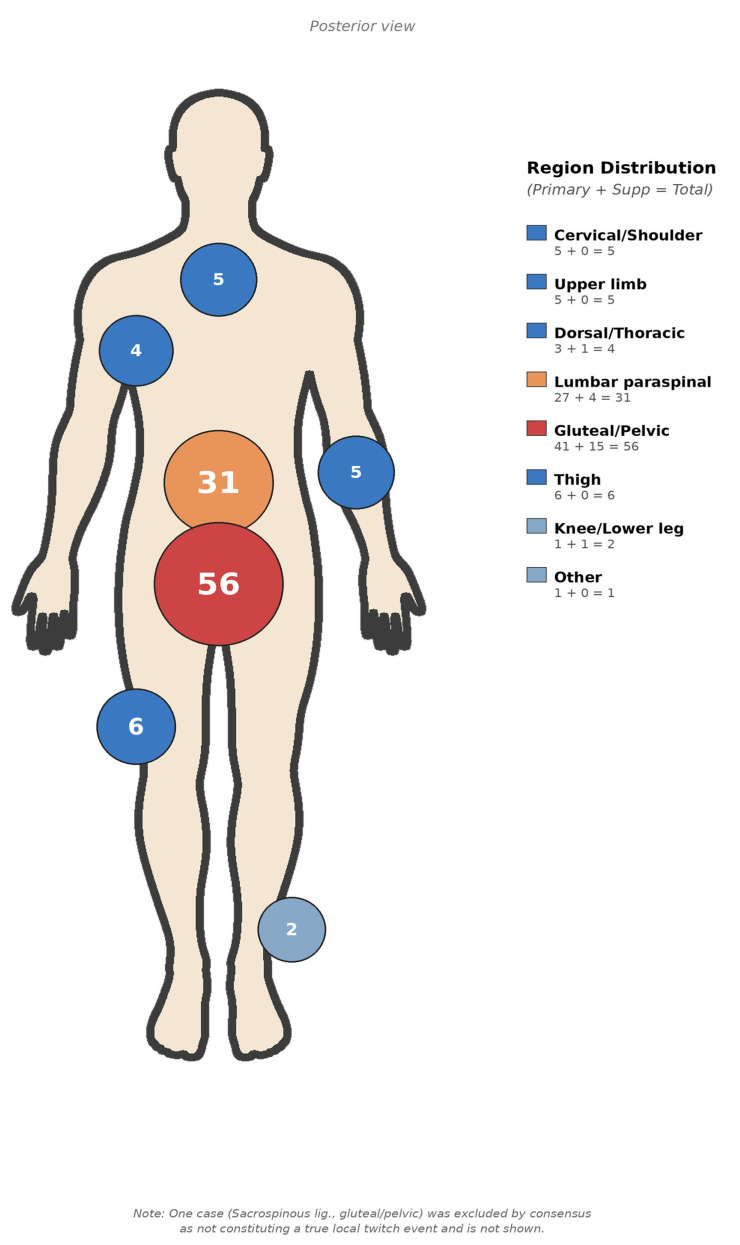
Anatomical distribution of the 110 confirmed local twitch events (89 primary-analysis + 21 supplementary-analysis). Circle size and color reflect total event count, with numbers within each circle indicating the total. The single excluded case (#22 sacrospinous ligament) is omitted from this figure (see Section 2.2 for details of this case). One additional case, originally labeled in our archive by patient identifier rather than by anatomical descriptor, was re-categorized to the lumbar paraspinal region during preparation of this revision (Section 2.3); the figure reflects the updated distribution (lumbar paraspinal n = 32).

Anatomically speaking, the 110 confirmed local twitches (89 primary plus 21 supplementary) were distributed across the gluteal/pelvic region (56, 50.9%), the lumbar paraspinal region (32, 29.1%; including one case originally labeled in our archive by patient identifier rather than by anatomical descriptor, re-categorized to the lumbar paraspinal region on the present re-review), the thigh (six, 5.5%), the cervical/shoulder region (five, 4.5%), the upper limb (five, 4.5%), the dorsal/thoracic region (four, 3.6%), and the knee/lower leg (two, 1.8%); the full anatomical distribution across all 110 confirmed events is shown in Figure 3. Across the 110 confirmed events (89 primary plus 21 supplementary), the most frequent specific sites were the obturator internus (10), piriformis (8), gluteus medius/minimus (7), gluteus maximus (7), sciatic nerve region (7), obturator externus (7), multifidus (6), quadratus lumborum (6), iliolumbar ligament (4), intertransverse ligament (3), sacrospinous ligament (2), and hamstring origin (2)—all anatomical locations at which stacking fascia is commonly encountered during ultrasound-guided FHR. The supplementary-analysis cohort (*n* = 21, unclear needle tip) showed a qualitatively similar distribution that was also dominated by the gluteal/pelvic (15/21, 71.4%) and lumbar paraspinal regions (4/21, 19.0%).

### 2.4. Secondary Outcomes: Quantitative Motion Characteristics

The computational screening pipeline is summarized in Figure 1, whose flowchart presents the overall cascade from the 11,205-video archive to the final confirmed cohort and underpins every Results subsection above. In numerical terms (see also Section 4.5), the first-stage Step A (image-quality and probe-stability filter) passed 4126 of 11,205 videos (36.8%). The second-stage Profile Match classifier subsequently extracted 417 candidates, which yielded the confirmed cohort described in Section 2.2 (111 events) after 30 visual-review rounds. The quantitative feature profiles of the 111 confirmed twitch events (Table 2) were consistent with the clinical observation. In particular, (a) the contraction duration had a median of 0.20 s (IQR 0.13–0.27, range 0.07–0.80), with all events lasting less than 1 s as single, brief contractions; and (b) the CB peak ratio (center-to-border motion ratio) had a median of 5.93 (IQR 4.82–8.24), objectively confirming that motion was localized to the ultrasound image’s central region (around the needle tip).

## 3. Discussion

This retrospective analysis of 11,205 ultrasound videos suggests that local twitches during ultrasound-guided FHR occur specifically at stacking fascia: in all 89 primary analysis cases with clearly visualized needle tips (after exclusion of one case reassessed on joint review as not a true twitch), the twitch was generated while the needle tip lay within stacking fascia (100%; initial independent inter-rater agreement, 95.6%). At the outset, because the FHR procedure is by design directed at stacking fascia, we acknowledge that this observation does not establish a positive predictive value for the structure independent of the intervention’s inherent targeting bias; rather, it documents the twitch-positive subset’s selectivity within an FHR cohort. We first consider the observational significance of this anatomical selectivity, then discuss the mechanistic candidates that could account for it, and finally place the findings within the broader context of ultrasound-guided FHR research.

### 3.1. Stacking Fascia as the Structural Substrate of Local Twitches

The 100% figure reported here should be interpreted as a characteristic of the twitch-positive subset—that is, an inclusion-criterion-bound selectivity—rather than as the positive predictive value of stacking fascia for twitch occurrence. Because FHR is by design directed at stacking fascia, this 100% figure is, in an important sense, tautological by intervention design; nonetheless, within the twitch-positive cohort, the anatomical locus is essentially homogeneous. The defining observation of this study is that local twitches, when they occur, are anatomically confined to stacking fascia. Of 89 primary analysis cases (after exclusion of one case reassessed as not a true twitch on joint re-examination), all 89 (100%) were generated while the needle tip lay within a layered, hyperechoic band-like structure consistent with stacking fascia. Importantly, this 100% selectivity (95% CI 95.9–100%) was reached after independent dual-rater determination (initial inter-rater agreement 86/90 = 95.6%) and adjudication of the four discordant cases by joint re-examination, thus strengthening the findings’ methodological credibility. The 100% figure was independent of whether the needle tip was at an extramuscular or intramuscular site; the structural feature consistently associated with twitch occurrence was the presence of stacking fascia. The clustering of events in regions where densified multilayered deep fascia is routinely encountered during FHR—the gluteal/pelvic region (51%) and lumbar paraspinal region (29%)—further supports stacking fascia as the phenomenon’s structural substrate. One important caveat applies: because our study design did not quantify how often the needle traverses non-stacking structures without producing a twitch, the 100% figure represents the twitch-positive subset’s selectivity rather than a direct estimate of the positive-predictive value of stacking fascia itself (Section 3.3). Within this bound, the observed 100% anatomical selectivity in the primary cohort suggests stacking fascia as the principal structural locus at which the local twitch phenomenon is concentrated.

A complementary observation deserves emphasis. Because FHR is, by design, directed at stacking fascia, the occurrence of twitches is in one sense expected; a more informative question is the converse—why, despite repeated needle passages through stacking fascia in routine practice, are local twitches so infrequently observed? Answering this question requires distinguishing “frequency” (per-procedure incidence, or the probability that a twitch is elicited per individual procedure) from “capture rate” (per-video archive rate, or the proportion of saved videos in which a twitch was recorded). Only the latter (0.98%, 110/11,205) can be computed from this study; the true per-procedure frequency is beyond the scope of this retrospective design. Two distinctions clarify the capture rate reported in this study. First, the operator (H.K.) viewed the ultrasound screen in real time throughout every procedure, so twitches were not missed during clinical observation. There were certainly cases in which a local twitch was clinically confirmed in real time on the ultrasound screen but was not captured in the preserved video archive (for example, because the recording button was not pressed within the 20 s pre-roll window); as the operator, the first author (H.K.), clinically observed local twitches more frequently than the rate captured in the preserved video archive, although a precise count of unrecorded clinically observed events was not maintained. Second, the per-video rate of 0.98% (110/11,205) reflects an archive of approximately 3% of the roughly 360,000 procedures performed during the study period, preserved on a clinically curated basis, and therefore, does not reflect the true clinical twitch occurrence rate per FHR procedure. The structural and biophysical conditions that determine whether contact with stacking fascia produces an observable twitch (i.e., the true per-procedure frequency) cannot be established with the present observational data, and their identification is thus left to future prospective work. The relation of the present observation to the classical trigger-point literature warrants comment. Travell and Simons [12] and Hong [13] described the ‘local twitch response’ as a brief muscle-band contraction elicited by needling, attributed to dysfunctional motor endplates within a taut band. The fascia-context twitch characterized here is not inconsistent with that classical framework; rather, it suggests that, within the FHR context, the structural locus most consistently engaged at the moment of twitch is the densified multilayered fascial interface (stacking fascia). Whether the same neurophysiological substrate (endplate activation) underlies both contexts, or whether mechanoreception by muscle spindles and other proprioceptive endings at the fascia-muscle interface contributes [10,11], is beyond what the present observational data can determine.

### 3.2. Clinical Implications

A practical implication follows directly from the previous observation. When observed within an FHR procedure, a local twitch provides a real-time ultrasound cue that the needle has reached stacking fascia—a moment-to-moment confirmation that the intended structural target has been engaged. Because twitches were captured in only a small fraction of FHR videos (0.98% in the present archive), their absence should not be interpreted as evidence that stacking fascia has been missed.

Several mechanistic hypotheses could account for the observed anatomical specificity. These include direct activation of fascia-resident mechanoreceptors and muscle-spindle afferents (including mechanosensitive Piezo ion channels [14]) at densified multilayered interfaces [10,11,12], mechanotransduction signaling via the YAP/TAZ pathway in fasciacytes and adjacent connective tissue cells [15], piezoelectric responses of densely packed collagen layers [16], active contractile properties recently described in deep fascia [7,17], and electrostatic phenomena across hyaluronic-acid-rich layers [3,4,5,6]. One specific biophysical hypothesis developed in detail elsewhere is the Fascial Capacitor Model [18]. We emphasize that the empirical findings reported here—the anatomical specificity of detected twitches to stacking fascia within the cohort—are observational and stand independently of any particular mechanistic model. Mechanistic interpretation lies beyond the scope of the present paper. Together with the previously published Fascial Memory Reset hypothesis [2], this paper contributes to a broader research program on ultrasound-guided FHR.

### 3.3. Limitations

This study has three principal limitations. First, the retrospective single-center design uses a clinically curated video archive rather than a random sample of all FHR procedures, and a 20 s pre-roll capture buffer privileged the moment of release rather than the moment of fascial contact. We acknowledge that this curation introduces a strong selection bias with respect to the underlying procedure population: the curation criterion (informative ultrasound visualization at the moment of recording) was applied prospectively and independently of any retrospective twitch detection, but was nonetheless not random. The per-video rate reported here (0.98%; 110/11,205) characterizes the curated archive and cannot be interpreted as the per-procedure incidence of local twitches in unselected FHR practice. Second, because the FHR procedure is by design directed at stacking fascia, the denominator of needle passages traversing non-stacking structures without producing a twitch was not quantified. The 100% figure therefore represents inclusion-criterion-bound selectivity within the twitch-positive subset, not positive predictive value for stacking fascia, and is in this sense tautological by intervention design. A controlled prospective design tracking all needle insertions—including those at non-stacking-fascia sites and those producing no twitch—would be required to establish PPV. Third, stacking-fascia adjudication was performed by two FHR-experienced clinicians, one of whom (H.K.) was the primary operator during the archive period. Independent verification by a blinded radiologist or sonographer without prior FHR involvement has not been performed in the present retrospective study. This reflects a structural feature of the early-stage characterization of a clinically specialized phenomenon: the ultrasound-visible local twitch response during FHR is a phenotype whose reliable identification depends on familiarity with the procedure, and at present the population of clinicians who combine high-resolution musculoskeletal ultrasound expertise with consistent prior recognition of this specific phenotype is essentially co-extensive with the small community of FHR-experienced clinicians. We anticipate that wider dissemination of FHR practice and of the present observational characterization will, over time, enable independent verification by clinicians whose ultrasound training postdates and is informed by—but is not itself part of—the originating research program. We acknowledge this as an inherent limitation of the present pioneering observational study. Because the cohort was assembled under the stacking fascia inclusion criterion (Section 4.4), the dual-rater review functions as verification of the inclusion criterion rather than as a classification task; Cohen’s kappa is therefore not applicable, and we instead report the raw dual-rater agreement (86/90 = 95.6%), together with full disclosure of the four discordant cases (Section 2.2). Consistent with the hypothesis-generating, observational character of this study (Section 4.6), the findings reported here are intended to motivate prospective work rather than to support inferential causal claims.

## 4. Materials and Methods

### 4.1. Study Design

This was a retrospective observational study designed as a two-site collaboration between Kimura Pain Clinic (Maebashi, Gunma, Japan) and Isesaki Municipal Hospital (Isesaki, Gunma, Japan), where one of the co-authors (T.H.) is affiliated. The study protocol was approved by the Ethics Committee of Isesaki Municipal Hospital (approval number: 2026-2) and covered both sites; this arrangement reflects standard practice in Japan when a single-physician outpatient clinic conducts research jointly with an institutionally affiliated senior collaborator: Isesaki Municipal Hospital served as the IRB-approving site (approval #2026-2; senior author T.H.’s affiliation), and Kimura Pain Clinic served as the data source (first author H.K.’s affiliation). As detailed below, no analyzable FHR ultrasound video recordings were obtained from Isesaki Municipal Hospital during the study period; the final analysis is therefore based on the 11,205 videos preserved at Kimura Pain Clinic. From 1 October 2016 onward, written informed consent was obtained at each patient’s initial visit; the consent form explicitly included permission for the ultrasound images, video recordings, and treatment course to be presented in academic meetings, journal publications, and books, on the understanding that no individually identifiable information would be disclosed. In addition, in accordance with the IRB-approved protocol, an opt-out procedure was implemented at both sites for patients who underwent ultrasound-guided FHR during the study period (1 October 2015 to 31 March 2026); however, no analyzable ultrasound video recordings of FHR procedures from Isesaki Municipal Hospital were available during the study period, and the final analysis was therefore based on the 11,205 videos preserved at Kimura Pain Clinic. A written notice describing the study’s purpose, the scope of data use (ultrasound video recordings preserved at the clinic), the anonymization procedure, the right to request exclusion without any disadvantage to clinical care, and the contact information of the lead investigator (T.H.) at Isesaki Municipal Hospital and of the data-providing facility (Kimura Pain Clinic) was posted at Kimura Pain Clinic from 18 April 2026 to 2 May 2026 (a two-week opt-out window). As of the date of manuscript submission, no patients had elected to opt out. Videos recorded before 1 October 2016 (approximately 2700 videos, representing about 24% of the total dataset) did not have individual prospective written consent and were therefore used retrospectively as pre-existing, de-identified clinical data under the IRB-approved opt-out framework described above. Ultrasound video files contain no personally identifying information (no patient names or facial images). During the study period, the first author (H.K.) performed approximately 360,000 ultrasound-guided FHR procedures (estimated as 150 procedures/day × 20 days/month × 12 months × 10 years). The 11,205 videos analyzed in this study correspond to approximately 3% of these procedures, and were preserved by the operator on a clinically curated basis when ultrasound visualization was judged to be informative. The video preservation criterion—perceived informativeness of ultrasound visualization—was applied prospectively at the moment of recording, and is independent of any retrospective twitch detection. The resulting per-video capture rate (0.98%; 110/11,205) therefore characterizes the curated archive rather than the underlying per-procedure incidence of local twitches.

### 4.2. Subjects

All cases of ultrasound-guided FHR performed at Kimura Pain Clinic between October 2015 and March 2026, for which ultrasound video recordings of the procedure had been preserved, were eligible (see Section 4.1 for consent details). A total of 11,205 ultrasound video recordings met the eligibility criteria. Cases for which the video quality was insufficient for analysis were excluded.

### 4.3. FHR Procedure

Ultrasound examinations were performed using a SONIMAGE HS2 portable ultrasound system (Konica Minolta, Inc., Tokyo, Japan), equipped with an L18-4 linear-array transducer (4–18 MHz) and a C5-2 convex-array transducer (2–5 MHz). Image acquisition was performed in B-mode using the system’s default musculoskeletal preset; representative on-screen acquisition parameters are reported in the Figure 1 caption. No external image-analysis software was used; ultrasound videos were reviewed in the manufacturer-supplied viewer, and the in-house Profile Match screening program (Python 3.11) described in Section 4.4 was used for computational candidate detection. FHR was performed under real-time ultrasound guidance using a 27-gauge needle. The needle insertion angle was selected by the operator according to the target anatomy and surrounding structures requiring avoidance; the approach typically lies on a continuum between strictly in-plane and strictly out-of-plane techniques. The needle-tip position was confirmed by direct ultrasound visualization when feasible (in-plane approach), and otherwise inferred from the characteristic tissue motion induced both by needle advancement and by the small-volume saline injections used to mark needle progression. For ultrasound acquisition, Simple Needle Visualization™ (SNV; Konica Minolta)—a proprietary multi-frame motion-detection algorithm that highlights moving structures (needle and injectate) as a blue color overlay on the B-mode image—was used. SNV does not rely on Doppler shift and therefore does not have an associated pulse repetition frequency (PRF), velocity scale, or color Doppler mechanical-index setting; instead, it operates by frame-to-frame analysis distinguishing moving (needle/injectate) from stationary (tissue) image components [19]. The procedure consists of identifying pathological fascial structures (stacking fascia) on ultrasound, before mechanically detaching and releasing the layered fascia by targeted needle insertion and saline injection. Whereas conventional hydrodissection and nerve block techniques primarily target neural structures, FHR is distinguished by its focus on the direct mechanical opening of pathological fascial layers visualized on ultrasound. Detailed technical descriptions of the FHR procedure—including a cadaver-validated study of injection spread—have been published previously [1,20,21]. When the operator considered an individual procedure to have informative ultrasound visualization (clear stacking fascia, well-resolved release dynamics, or other clinically illustrative features), an approximately 20 s video was preserved using the ultrasound system’s pre-roll buffer feature: pressing the record button captured the preceding 20 s of imaging. The decision to record was typically made after the release was complete; videos were therefore preserved on a clinically curated basis, rather than for every procedure performed.

### 4.4. Video Analysis and Outcomes

For operational purposes, a local twitch was defined as a brief, single involuntary contraction (lasting less than 1 s) observed on ultrasound imaging at the moment the needle tip contacted a fascial layer during FHR, involving the fascial layer at the needle–tissue interface and immediately adjacent muscle. The 1 s threshold was based on empirical contraction duration measurements from an independently collected reference set of 17 representative twitch videos (frame-difference half-maximum analysis; median 0.27 s, range 0.13–0.93 s, all under 1 s). Stacking fascia was defined as a layered, hyperechoic band-like structure observed on high-resolution ultrasound (Konica Minolta; linear probe L18-4, 4–18 MHz; convex probe C5-2, 2–5 MHz).

Primary outcome: The primary outcome was whether the needle tip was at stacking fascia at the moment of local twitch occurrence (yes/no). The proportion of twitch events occurring at stacking fascia (with 95% Clopper–Pearson confidence interval) was the principal quantity of interest.

Secondary outcomes: Secondary outcomes were (a) the anatomical distribution of twitch events across body regions; (b) the per-video rate of captured twitch events within the 11,205-video archive; (c) the inter-rater agreement of stacking fascia determination (independent dual-rater raw agreement with full disclosure of discordant cases; Cohen’s kappa is not applicable under this hypothesis-verification design); and (d) the quantitative motion characteristics of confirmed twitches (spike width, center-to-border ratio, and Profile Match score) derived from the auxiliary frame-difference analysis (Section 4.5).

Local twitch events were identified in two complementary phases. In the clinical observation phase, one author (H.K., with more than 10 years of experience with ultrasound-guided FHR) identified local twitch events prospectively during routine FHR practice from 2015 through 2026. In the computational-screening phase, a frame-difference algorithm (Profile Match classifier; see Section 4.5) was applied to all 11,205 available ultrasound videos to systematically detect events that may have been missed during routine clinical practice. The 417 candidates flagged by the algorithm were visually reviewed by H.K. over 30 review rounds. Taking the two phases together, a total of 111 local twitch events were initially confirmed. Of these, 21 cases in which the position of the needle tip could not be clearly identified on the ultrasound image were assigned to a supplementary analysis, and the remaining 90 cases with a clearly visualized needle tip constituted the initial primary analysis set. After independent dual-rater determination of stacking fascia status at each needle-tip site (Section 4.6), one case in the primary analysis set (sacrospinous ligament site) was reassessed by consensus on joint re-examination as not constituting a true local twitch event (likely voluntary muscle movement) and was excluded.

For each twitch the following parameters were recorded:

(a) Presence or absence of a local twitch;
(b) Time of twitch occurrence within the video;
(c) Anatomical site (lumbar, gluteal, thigh, cervical, etc.);
(d) Presence or absence of stacking fascia at the needle-tip site, defined as a layered, hyperechoic band-like structure on ultrasound (an ultrasound phenotype of densified multilayered fascia, which can occur at either extramuscular or intramuscular locations).

### 4.5. Auxiliary Quantitative Analysis

The computational screening consisted of a two-step pipeline. The physical meaning, definition, and threshold of each metric are described below. Basic principle: ultrasound videos consist of consecutive frames; inter-frame pixel-difference extracts regions where motion occurred between frames (frame-difference method). A local twitch manifests as a brief (under 1 s), localized motion around the needle tip; therefore, the algorithm combines (i) spatial localization of motion, (ii) temporal sharpness of motion, and (iii) overall recording stability. Step A (primary motion filter): each video was resampled at 10 frames per second (FPS) with a 0.5× downscale, and inter-frame pixel differences were computed. The frame was divided into a 6 × 6 grid, with the inner 4 × 4 designated as the center region (around the needle tip) and the outer ring as the border region (image edges). Five criteria were applied sequentially: mean correlation ≥ 0.96 (recording stability: correlation between consecutive frames, closer to 1 indicates a more stable probe); Doppler ratio < 1.05 (excludes color-Doppler frames); peak isolation: peak_fraction < 0.15 (temporal spike isolation; ensures the twitch is single and brief); center sigma ≥ 2.0 (intensity of central motion: large values indicate strong local contraction); and border/center ratio < 0.40 (spatial localization: smaller values indicate motion concentrated at the center). Step B + Profile Match classifier: candidates passing Step A were re-analyzed at the native frame rate (15–30 FPS) with the following additional criteria: spike-width 2–15 frames (full-width at half-maximum of the motion peak, corresponding to contraction duration, approximately 0.07–1.0 s at native FPS); CB peak ratio ≥ 3.0 (center-to-border motion ratio: larger values indicate greater locality); and profile variance ≥ 500 (variance of the spatial profile: selects videos in which fascial layer boundaries are well visualized). Candidates were ranked by combined primary filter score (profile_var × cb_peak/1000). Interpretation of each metric: spike-width reflects contraction duration (shorter = more twitch-like); CB peak ratio reflects locality (higher = more local); center sigma reflects intensity of central motion; mean correlation reflects recording stability (closer to 1 = more stable); combined score reflects overall confidence that the event is a true twitch (higher = greater confidence). Quantitative ranges of each metric and their interpretation are summarized in Table 2. The Step B threshold of spike-width ≥ 2 frames was chosen to balance detection sensitivity against manual review burden: events with spike-width = 1 frame, while clinically observable, would have produced an excessive number of false-positive candidates from probe micro-motion and other noise. Such brief events were captured through the parallel prospective clinical observation phase (Section 4.4).

### 4.6. Statistical Analysis

**Figure 4:**
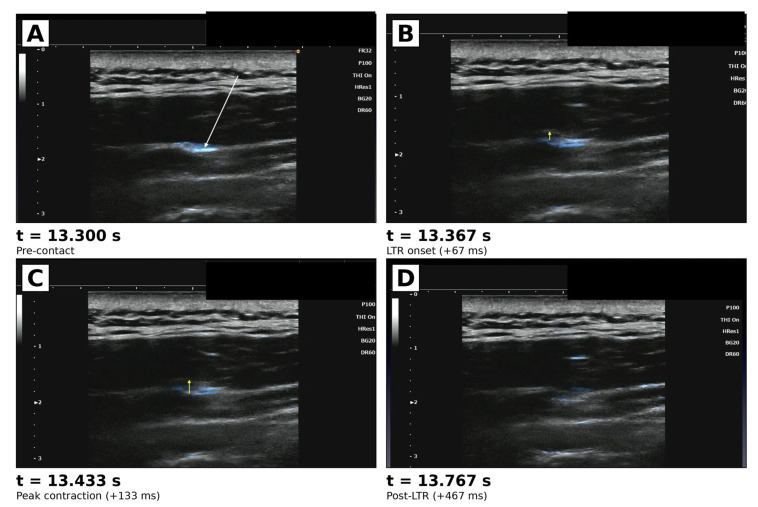
Frame-by-frame ultrasound visualization of a local twitch response (LTR) during ultrasound-guided fascial hydrorelease (FHR), recorded in the trapezius/rhomboid region. (**A**) Pre-contact (t = 13.300 s): the 27-gauge needle tip approaches the stacking fascia. The needle itself is not directly visible; needle-tip position is inferred from the characteristic tissue motion induced by needle advancement and by small-volume saline injection, visualized via Simple Needle Visualization™ (SNV; Konica Minolta)—a proprietary multi-frame motion-detection algorithm that overlays needle and injectate motion as a blue color signal on the underlying B-mode image (cf. [20,19]). (**B**) LTR onset (t = 13.367 s; +67 ms): the LTR begins at the moment the needle tip contacts the stacking fascia. The yellow arrow indicates the direction of the upward contraction. (**C**) Peak contraction (t = 13.433 s; +133 ms): maximum tissue displacement during the LTR. Contraction involves the stacking fascia and adjacent muscle. (**D**) Post-LTR (t = 13.767 s; +467 ms): tissues return to a relaxed state. The saline spread appears more dispersed than in (**A**), reflecting tissue displacement during contraction. In the present retrospective cohort of 11,205 ultrasound videos recorded during FHR, 100% of 89 final primary analysis local twitches (after independent dual-rater determination with adjudicated consensus) were generated while the 27-gauge needle tip lay within stacking fascia, whereas no local twitches were observed when the needle tip lay outside stacking fascia in the primary cohort. Local twitch is a brief (less than 1 s) involuntary contraction observed at the moment the needle tip contacts the stacking fascia, involving the stacking fascia and adjacent muscle. SNV is not a Doppler-based modality and therefore does not have an associated pulse repetition frequency, velocity scale, or color-Doppler mechanical-index setting; it operates by frame-to-frame motion analysis distinguishing moving (needle/injectate) from stationary (tissue) image components [19].

Descriptive statistics were used to summarize local twitch frequency, anatomical distribution, and proportion at stacking fascia. Stacking fascia determination was performed independently by two reviewers (H.K. and T.H.) by visually inspecting the ultrasound images at the moment of each twitch event, evaluating the needle tip and the adjacent fascial layer; the second reviewer (T.H.) was blinded to the first reviewer’s determination at the time of independent assessment. Discordant cases were resolved by joint re-examination of the relevant ultrasound video clips, with both reviewers sharing their reasoning until consensus was reached. Initial inter-rater agreement on the primary analysis cohort prior to consensus discussion was 86 of 90 (95.6%; 95% CI 89.0–98.8%); the four discordant cases were resolved as described in Section 2.2 (one excluded as not constituting a true twitch event; three confirmed at stacking fascia upon joint re-examination). Because the cohort was assembled by purposive sampling under the stacking fascia inclusion criterion (Section 4.4), the dual-rater review functions as verification that each event meets this inclusion criterion, rather than as a discrimination task between heterogeneous classes. Under this design, Cohen’s kappa is not an appropriate index of inter-rater reliability—as its underlying assumption of substantive variance in true classes is not met—and we therefore report the raw dual-rater agreement together with full disclosure of all discordant cases (Section 2.2) as a transparent record of the verification step. The 95% confidence intervals for proportions were calculated by the Clopper–Pearson exact method. The overall twitch incidence was reported as a percentage. Because this study is a hypothesis-generating observational study, inferential statistical testing was not used as a primary analytic strategy. A representative time-resolved example of a single LTR event is illustrated in Figure 4.

## 5. Conclusions

In a retrospective analysis of 11,205 ultrasound videos recorded during ultrasound-guided FHR, all 89 primary analysis local twitches were clearly visualized and occurred while the needle tip lay within stacking fascia (89/89, 100%; 95% CI 95.9–100%). Events were predominantly concentrated in the gluteal/pelvic (51%) and lumbar (29%) regions. The principal observation of this study is the anatomical relationship between local twitch occurrence and stacking fascia status at the needle tip; mechanistic interpretations are beyond the scope of this observational paper.

## Figures and Tables

**Table 1:** Ultrasound video dataset characteristics.

Characteristic	Value
Study period	October 2015–March 2026 (~10 years 6 months)
Sites included	Kimura Pain Clinic (Approved data source); Isesaki Municipal Hospital (Ethics approval site, no archived videos available)
Total ultrasound-guided FHR video recordings, n	11,205
Recorded with written informed consent (on or after 1 October 2016)	8505 (~76%)
Used as de-identified pre-existing clinical data (before 1 October 2016)	2700 (~24%)
Mean recording duration, s	~20
Ultrasound device	Konica Minolta SONIMAGE HS
Probes used	L18-4 (linear, 4–18 MHz); C5-2 (convex, 2–5 MHz)
Confirmed local twitch events, n (%)	111 (0.99%)
Primary-analysis set (clearly visualized needle tip)	90
Supplementary analysis (unclear needle tip)	21
Candidates flagged by Profile Match classifier, n	417
Rounds of consensus visual screening	30

Note: FHR—fascial hydrorelease. The 111 confirmed events represent the count before consensus re-examination; after the exclusion of one case (#22, sacrospinous lig.) by joint review, the final confirmed cohort consisted of 110 events (89 primary + 21 supplementary; see Section 2.2). The study was approved as a multi-site collaboration between Kimura Pain Clinic and Isesaki Municipal Hospital, with opt-out implemented at both sites; however, no analyzable ultrasound video recordings of FHR procedures from Isesaki Municipal Hospital were available during the study period, and the final analysis was therefore based on videos preserved at Kimura Pain Clinic. Recording periods straddling the consent transition (1 October 2016) were handled as specified in Section 4.1. Background colors used in this table: the blue header band identifies the column headings; indented sub-rows further break down the immediately preceding parent row (consent framework: written informed consent vs. pre-existing data; confirmed twitch events: primary-analysis vs. supplementary subsets).

**Table 2:** Quantitative feature profiles of 111 confirmed local twitch events (frame-difference measurements).

Metric	Median	IQR	Range	Physical Meaning
Duration (s)	0.20	0.13–0.27	0.07–0.80	Contraction duration
Spike width (frames)	3	2–4	1–12	Full-width at half-maximum of motion peak
CB peak ratio	5.93	4.82–8.24	3.38–77.77	Center-to-border motion ratio (locality)
Center peak intensity	10.69	8.14–19.21	4.38–138.94	Intensity of central motion

Note: Frame-difference analysis was applied to all 111 confirmed twitch videos (89 primary + 21 supplementary + 1 consensus-excluded). Native frames per second (FPS)—15 were used. The 17 events identified during the prospective clinical observation phase (Section 4.4) were captured by direct visual recognition by the operator and did not pass through the Step B threshold (spike width ≥ 2 frames), accounting for the lower-bound spike width of 1 frame in this table. Metric definitions are provided in Section 4.5.

## Data Availability

The data presented in this study are available on request from the corresponding author. The data are not publicly available due to patient privacy restrictions under the IRB-approved opt-out framework (Isesaki Municipal Hospital, approval number 2026-2).

## Supplementary Materials

The following supporting information can be downloaded at https://www.mdpi.com/article/10.3390/medsci14030350/s1, Supplementary Video S1: Local twitch in the gluteus maximus/semimembranosus (corresponding to Figure 2). Localized contraction at the moment the needle tip contacts a fascial layer during FHR (MP4, 15 fps, approximately 20 s). Supplementary Video S2: Local twitch in the trapezius/rhomboid. Localized contraction at the moment the needle tip contacts a fascial layer during FHR (MP4, 15 fps, approximately 13 s). Supplementary Video S3: Local twitch in the iliocostalis (posterior aspect). Localized contraction at the moment the needle tip contacts a fascial layer during FHR (MP4, 15 fps, approximately 13 s).

## Abbreviations

FHR	Fascia Hydrorelease (also fascial hydrorelease)
LTR	Local Twitch Response
CI	Confidence Interval
IQR	Interquartile Range
FPS	Frames Per Second
FR	Frame Rate
THI	Tissue Harmonic Imaging
HRes	High Resolution
BG	Brightness Gain
MI	Mechanical Index
TIS	Thermal Index for Soft Tissue
TIB	Thermal Index for Bone
CB	Center-to-Border (peak ratio)
YAP	Yes-Associated Protein
TAZ	Transcriptional Coactivator with PDZ-binding Motif
IRB	Institutional Review Board
JNOS	Japanese Non-surgical Orthopedics Society
